# Dubious decision evidence and criterion flexibility in recognition memory

**DOI:** 10.3389/fpsyg.2015.01320

**Published:** 2015-09-08

**Authors:** Justin Kantner, Jean M. Vettel, Michael B. Miller

**Affiliations:** ^1^U.S. Army Research Laboratory, Aberdeen, MDUSA; ^2^University of California, Santa Barbara, Santa Barbara, CAUSA

**Keywords:** recognition, decision making, criterion shifting, response bias, feedback

## Abstract

When old–new recognition judgments must be based on ambiguous memory evidence, a proper criterion for responding “old” can substantially improve accuracy, but participants are typically suboptimal in their placement of decision criteria. Various accounts of suboptimal criterion placement have been proposed. The most parsimonious, however, is that subjects simply over-rely on memory evidence – however faulty – as a basis for decisions. We tested this account with a novel recognition paradigm in which old–new discrimination was minimal and critical errors were avoided by adopting highly liberal or conservative biases. In Experiment 1, criterion shifts were necessary to adapt to changing target probabilities or, in a “security patrol” scenario, to avoid either letting dangerous people go free (misses) or harming innocent people (false alarms). Experiment 2 added a condition in which financial incentives drove criterion shifts. Critical errors were frequent, similar across sources of motivation, and only moderately reduced by feedback. In Experiment 3, critical errors were only modestly reduced in a version of the security patrol with no study phase. These findings indicate that participants use even transparently non-probative information as an alternative to heavy reliance on a decision rule, a strategy that precludes optimal criterion placement.

## Introduction

Decision making is often guided by bias, and bias is often adaptive. Indeed, the ability to take the same action freely in some situations and cautiously in others is essential to decision making in everyday life. For example, an individual must shift from a more accepting to a more skeptical stance in evaluating information from more or less reputable sources; a student readily answers questions while among friends, but is extremely cautious in a classroom setting; a basketball player passes on a shot while protecting a lead, but not when trying to catch up. Such criterion shifts – between liberal and conservative standards of evidence for a decision – tailor decision strategy to the situation and can be essential to avoiding errors, especially when decision evidence is ambiguous.

Criterion shifting has been of substantial interest in recognition memory (Hockley, 2011), a task domain well suited to studying the interaction of memory and decision processes. According to most models of recognition, judgments as to whether a person, place, or object has been encountered previously are based on whether its appearance elicits a criterial level of memory evidence (Parks, 1966). One can use (1) a liberal criterion, accepting items as old on the basis of relatively little memory evidence, (2) a conservative criterion, requiring substantial evidence before making an “old” judgment, or (3) a relatively neutral criterion, favoring neither response. When memory evidence is ambiguous, context-appropriate criterion shifting can produce accurate decisions. Consider the case of meeting a person who strikes us as vaguely familiar: if the encounter occurs during a vacation, where we would not expect to see many people we know, we should use a conservative decision criterion and conclude that the individual is new. If it occurs in our neighborhood, by contrast, we should be more likely to conclude that we know the individual.

Despite the adaptive value of criterion shifting for decision making, recognition memory studies demonstrate that individuals are limited in their ability (or inclination) to make such shifts. There is little doubt that participants can make appropriate shifts under some conditions: provided with corrective feedback and/or explicit instructions, participants can adapt bias to within-list changes in the prior probability of an old item (e.g., Rhodes and Jacoby, 2007; Aminoff et al., 2012) and the memory strength of old items (Verde and Rotello, 2007; Singer, 2009). They can also utilize a more liberal criterion for items tested after a long delay than after a short delay (Singer and Wixted, 2006), a more conservative criterion when distractors are highly similar to targets than when they are dissimilar (e.g., Benjamin and Bawa, 2004), and a more conservative criterion for recognizing familiar than unfamiliar scenes (Dobbins and Kroll, 2005). In the absence of feedback or highly salient differences between two item classes, however, criterion shifts are generally not observed (e.g., Rhodes and Jacoby, 2007; Verde and Rotello, 2007). Thus, research has investigated the circumstances that do versus do not elicit criterion shifts in order to determine the flexibility of the recognition system to change decision rules.

Although much research has examined the question of when criterion shifts occur, less is known about why they are often found to be inadequate to maximize accuracy or expected payoffs (e.g., Ulehla, 1966; Benjamin et al., 2009; Aminoff et al., 2012). A criterion that maximizes a recognizer’s proportion of correct responses must be calibrated both to the conditions of the task and to the individual’s ability to discriminate old items and new (see Green and Swets, 1966). If participants are told that 70% of test items are old, for example, an “old” response should be given whenever the recognizer is unsure; individuals with lower recognition sensitivity are unsure on a higher proportion of trials than those with greater sensitivity, and should set more liberal criteria to improve their percentage of correct responses [for further discussion of this point see Lynn and Barrett (2014)]. If old–new discrimination is at chance, for example, one should respond “old” on every trial, a strategy that would result in 70% accuracy. Aminoff et al. (2012) tested shifting between blocks containing 70 and 30% old items and used each participant’s sensitivity (*d’*) score to calculate the criterion with which s/he would maximize proportion correct. Though they found substantial individual differences in the magnitude of shifts, no participant shifted widely enough to maximize accuracy.

Researchers have advanced several hypotheses regarding the basis of inadequate criterion shifting (also called “conservatism”). Ulehla (1966, p. 569) noted that “subjective probability lags behind objective probability” in some choice domains and proposed that participants in signal detection tasks over/underestimate target base rates. Parks (1966) noted that a strategy of distributing “old” and “new” responses according to the base rates (i.e., “probability matching”) produced insufficient criterion placement, and Thomas and Legge (1970) reported data suggesting that participants do use such a strategy (though subsequent work disfavored probability matching as a full account of criterion placement; Thomas, 1975; Benjamin et al., 2009). Kubovy (1977) suggested that suboptimal criterion placement is driven by incorrect intuition as to the shape of the target and lure evidence distributions. Benjamin et al. (2009) presented evidence that trial-by-trial noise in the decision criterion – a consequence of the effort required to maintain and shift the criterion – can produce conservatism in criterion shifting when base rates are manipulated.

Also relevant to the question of inadequate criterion shifting are general models of criterion placement. According to the means model (Hintzman, 1994), participants estimate the mean of the “old” item distribution, perhaps by learning the average increment in memory strength afforded by study list presentation, and establish a criterion at a point between this “old” mean and the mean of a pre-experimentally familiar new-item distribution. According to the range model (Parducci, 1984), participants estimate the highest and lowest memory strength values to be encountered at test, perhaps by assessing the memory strength of easily recalled old items and new items, respectively, and establish their criterion between these endpoint values. Hirshman (1995) tested these two models in addition to one based on the probability matching strategy described above; his analyses weighed in favor of the range model. Recently, Lynn and Barrett (2014) outlined a “utilized” signal detection framework for modeling optimal criterion placement according to three environmental factors: target base rates, the costs, and benefits (financial or otherwise) associated with decision outcomes, and the similarity of targets to lures. According to this model, suboptimal criterion placement results from a failure to properly estimate one or more of these variables.

While any of the above mechanisms may help explain criterion placement for a given participant and/or a given criterion manipulation, an additional hypothesis with considerable explanatory reach has received relatively little attention. In their seminal treatment of signal detection theory, Green and Swets (1966) proposed that participants fail to place sufficiently extreme criteria because they are unwilling to abandon the use of decision evidence, even when that evidence leads to uncertainty. Green and Swets (1966) described this phenomenon as follows:

“The observer tends to avoid extreme criteria: when the optimal β is relatively large, his actual criterion is not so high as the optimal criterion, and when the optimal β is relatively small, his criterion is not so low as the optimal criterion. Although this pattern is consistent with studies of decision making under uncertainty which do not involve ambiguous sensory information, the significance of its appearance here is not totally clear. It may be suspected that the subject’s natural disinclination to make the same response on all trials is strengthened by his awareness that the experimenter’s principle interest is in a sensory process. He probably finds it difficult to believe that he would be performing responsibly if the sensory distinctions he makes are exactly those that he could make by removing the earphones in an auditory experiment or by turning his back on a visual signal. (p. 91)”

As Green and Swets noted, participants are aware that the experimenter is interested in a sensory process, a fact that may limit their willingness to abandon sensory/memory evidence in making judgments. To the extent that participants prefer to base recognition decisions on their own resolution of an ambiguous signal rather than defer to a decision rule (e.g., “When most of the items are old and I am unsure, I should respond “old”), their criterion will be suboptimal. This account is a powerful one in that it can be applied to any of the commonly used criterion manipulations (i.e., base rates, payoffs, instructional motivation). In addition, it avoids assumptions of potentially taxing mental computations such as target probabilities, response frequencies, or features of the distributions that subjects may be unable or disinclined to perform.

Despite its generality and relative simplicity, the hypothesis that inadequate criterion shifting is driven by an overreliance on memory evidence (and a consequent under-reliance on probative non-memory forms of evidence, such as payoffs or base rates) has gone largely unexamined. The present experiments were designed to test this hypothesis by assessing the extent of participants’ reliance on decision evidence in recognition memory. In a departure from standard recognition procedures, we tested the effects of three criterion manipulations using an extremely homogeneous stimulus set that yielded near-zero old-new discrimination, such that participants could easily perceive the ambiguity of the memory evidence. The magnitude of criterion shifts was measured as inverse evidence of reliance on this dubious memory evidence: to the extent that participants resist basing decisions on memory evidence, they should follow the decision rule mandated by the criterion manipulation and adopt extreme conservative/liberal criteria. To the extent that they avoid setting extreme criteria, we infer that they are using transparently non-probative evidence to make recognition decisions. Across three experiments and three types of criterion manipulation, our results suggest that the use of such evidence is frequent and a major influence on recognition decisions, whether memory evidence is entirely ambiguous (Experiments 1 and 2) or altogether absent (Experiment 3).

## Experiment 1

The design of Experiment 1 varied from that of standard recognition memory paradigms in two respects. First, recognition experiments are typically designed such that participants have at least moderate old-new discrimination at test. The more participants are able to use memory evidence to make decisions, the less they benefit from criterion shifting. Additionally, even modest levels of discrimination may be sufficient for participants to *believe* that they can rely on memory evidence and eschew large shifts (Aminoff et al., 2012). To create a strong test of participants’ use of memory evidence, we used an extremely homogeneous stimulus set (described below) in which old and new items are so similar that discrimination is near chance. Such a transparently difficult recognition task should lead subjects to place minimal weight on memory evidence and yield to a decision rule as a basis for memory decisions.

A second feature of most recognition experiments is that the impetus for making accurate judgments is simply accuracy itself, except when financial incentives are used (e.g., Healy and Kubovy, 1978). Perhaps individuals can readily be induced to limit their use of memory evidence when the recognition task provides a compelling subjective reason to bias responses. We created such a task by converting a typical recognition paradigm into a “security patrol for suspicious persons.” Participants studied a list of “suspicious” individuals and, at test, were told to respond “suspicious” to anyone recognized from the study list and “innocent” to anyone not on the study list. In liberal blocks, participants were informed that calling an individual “suspicious” meant pulling that individual aside for questioning and search, and participants should respond “suspicious” whenever in doubt. In conservative blocks, calling an individual “suspicious” meant that they would be subject to “aggressive pursuit, probable injury, and capture,” and participants should respond “innocent” whenever unsure. To ensure that criterion shifts were motivated solely by the shifts in the nature of the patrols, the base rate of targets was fixed at 0.30 across blocks (a proportion that produced a minority of suspicious individuals but left enough target trials to allow reliable estimates of sensitivity and response criterion). Coupled with the extremely poor discrimination of suspicious and innocent probes, the importance placed on avoiding critical errors in the two types of patrols (see Method for full instructions) should provide an abundance of incentive to adopt extreme response criteria in both liberal and conservative blocks, shifting widely between the two scenarios.

As a comparison condition, we also tested shifting in a standard target probability manipulation. In the liberal (conservative) test blocks, 70% (30%) of test items were old; the stimuli were those used in the security patrol condition, but no mention of a patrol scenario was made. Each participant completed both tasks (Percent and Patrol) to enable within-subjects comparisons of criterion shifting, critical misses, and critical false alarms across different sources of motivation.

Finally, to further motivate criterion shifting, half of the participants in each group received trial-by-trial feedback at test. Feedback was tailored to each task (see Method) and not only conveyed the accuracy of responses but, in the case of errors, provided persistent reminders of the appropriate bias in a given test block.

### Method

#### Participants

One hundred twelve undergraduates at the University of California, Santa Barbara participated for course credit. The feedback and no feedback conditions included 57 and 55 participants, respectively. All experiments reported in this article were approved by the Human Subjects Committee at the University of California, Santa Barbara.

#### Materials

Stimuli were 324 full-body human models created with 3ds Max software (Autodesk, Inc). Each item combined a unique head model with one of six hairstyles (created with FaceGen, Singular Inversions Ltd.) and one of eight body models (ES3DStudios) wearing one of six clothing styles in one of 13 clothing colors. Thus, each individual had a unique face, but non-face features overlapped. Half of the models were male.

**FIGURE 1:**
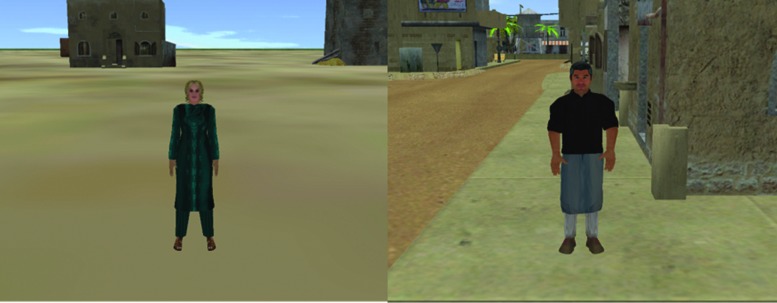
**Examples of stimuli used in the current experiments: female with outskirts background **(left)** and male with city background (right)**.

Each model appeared against a white background for presentation during study and embedded in a realistic desert environment (ES3DStudios) for presentation at test. Sixteen scenes were used; half depicted the center of a city and half depicted the outskirts. Individual models were centered in each scene and faced forward. Test probes were presented against city backgrounds during one type of patrol and against outskirts backgrounds during the other (counterbalanced across participants). Thus, the backgrounds provided a visual context consistent with a liberal “city patrol” and a conservative “outskirts patrol,” or vice versa. Examples of the stimuli appear in **Figure 1**.

Study and test lists for each of two recognition study-test cycles were created via random selection from the 324-item pool for each participant. The study list for each cycle was composed of 70 randomly selected individuals appearing against white backgrounds. The test lists for each cycle included a randomly ordered intermix of studied and non-studied individuals, each appearing against a city or outskirts background. No items were repeated between the two study-test cycles. The experiment was conducted with E-Prime software (Psychology Software Tools, Inc., Sharpsburg, PA, USA).

#### Procedure

Each participant completed two recognition study-test cycles, one a Percent task (target probability manipulation) and the other a Patrol task. Half of the participants received trial-by-trial feedback and half received no feedback. In each task, 70 items were studied for 2 s each with a 1-s inter-stimulus interval. Tests were divided into four 35-item blocks that interleaved liberal and conservative conditions. The assignment of tasks and feedback conditions to participants, task order, test block order, assignment of city/outskirts backgrounds to liberal/conservative patrols, and ordering of the both study and test lists were randomized anew for each participant.

#### Patrol Task

Participants were informed that they would be taking part in a simulated security patrol for suspicious persons and that they would begin by studying suspicious individuals to be recognized later. Test instructions informed participants that they would be presented with a mixture of suspicious people from the study list and innocent people they had never seen and that their task was to respond “suspicious” (by pressing the “1” key) to the former and “innocent” (by pressing “0”) to the latter. Participants were told that the nature of the patrol would vary according to the location of the individuals. For liberal blocks, participants received these instructions:

“While you are on patrol in the outskirts, identifying an individual as SUSPICIOUS means that individual will be pulled aside for questioning and search. It is VERY IMPORTANT not to miss any of the suspicious people you saw earlier. Those people are potentially dangerous and need to be questioned and searched. Remember, in the outskirts, you definitely do not want to miss any of the suspicious people you saw earlier. It’s fine if you mistakenly pull aside some people for questioning who turn out to be innocent (not seen before). This is a minor inconvenience for them. But make sure you don’t miss any of the suspicious people you saw earlier!”

For conservative blocks:

“While you are on patrol in the city, identifying an individual as SUSPICIOUS means that they will be hunted down like a dangerous criminal. This will include aggressive pursuit, probable injury, and capture to the people that you identify. It is VERY IMPORTANT not to mistakenly identify an innocent person as suspicious. It would be an injustice to subject an innocent person to this treatment. Remember, in the city, you definitely do not want to identify innocent people as suspicious. It’s fine to miss some people who turn out to be suspicious (that you saw before). It is expected that some suspicious people will escape capture on this patrol. But make sure you don’t mistakenly hunt innocent people!”

The eight city and eight outskirts locations were introduced for 3 s each, accompanied by the type of error to be avoided (misses in liberal blocks and false alarms in conservative blocks). Each test block was characterized as a city patrol or an outskirts patrol. The base rate of old items was 0.30 in each block. Instructions preceding each block reminded participants of the crucial importance of avoiding critical misses or false alarms. Test responses were non-speeded. A blank 2-s interval separated each trial.

#### Percent Task

Participants were informed that they would be studying a list of individuals to memorize. Test instructions explained that city or outskirts location was diagnostic of probability old. The locations were introduced alongside the corresponding base rate of old items. Test blocks were defined by the prior probability of an old item (70 or 30%). Instructions appeared before each test block reminding participants of the percentage of old items in that block and explicitly advising that because most of the individuals in the block were (were not) on the study list, one should respond “old” (“new”) whenever unsure.

#### Feedback

Except for the presence of trial-by-trial feedback at test and related instructions, the feedback and no feedback versions of the tasks were identical. Instructions stated that feedback would be presented after each response and encouraged participants to use it to improve their decisions. In the Percent task, correct answers were followed with the phrase “Correct! That individual was/was not studied!” in a blue font. In the Patrol task, feedback read “Correct! That individual was suspicious/innocent!” in a blue font.

Feedback to incorrect responses varied between liberal and conservative blocks. In liberal blocks, Percent feedback was “Okay, but that individual was not studied” in a black font following a false alarm and “Wrong! That individual was studied! Remember, 70% are OLD!” in a red font following a miss. Patrol feedback read “Okay, but that individual was innocent” following a false alarm and “Wrong! That individual was suspicious! Remember, don’t miss anyone SUSPICIOUS!” following a miss. Analogous feedback was given in the conservative blocks. Feedback was presented for 2 s.

### Results and Discussion

Recognition sensitivity (*d′*) was calculated as z(H) – z(FA), where H and FA are the hit and false alarm rates, respectively. Response bias was measured with *c*, equal to –[z(H) + z(FA)]/2. Hit and false alarm rate values of 0 and 1 were adjusted via Macmillan and Kaplan’s (1985) method to enable calculation of *d*′ and *c*: rates of 0 were adjusted upward to 0.5/N and rates of 1 were adjusted downward to 1-(0.5/N), where N is the number of signal trials (for hit rates) or the number of noise trials (for false alarm rates).

The mean *d*′ and *c*-values in each condition appear in **Table 1**. As expected, old–new discrimination was very poor. Across the patrol and percent tasks, the mean *d*′ for all participants was 0.132 (corresponding to a mean hit rate of 0.51 and a mean FA rate of 0.46). This value was significantly greater than zero, *t*(111) = 7.925, *p* < 0.001, indicating minimal but statistically above-chance discrimination. *d*′ data were analyzed with a 2 (Task: Percent vs. Patrol) × 2 (Bias: Liberal vs. Conservative) × 2 (Feedback: Present vs. Absent) mixed factor ANOVA with Task and Bias as within-subjects factors and Feedback as a between-subjects factor. *d*′ scores were modestly but significantly higher during liberal test blocks (*M* = 0.177) than during conservative test blocks (*M* = 0.096), *F*(1,110) = 8.026, *p* < 0.01, $\eta_{p}^{2}$ = 0.068, but did not vary as a function of task (*p* = 0.62) or feedback (*p* = 0.26). There were no significant interactions (all *p*s > 0.62).

**Table 1:** Mean *d*′ and criterion values in liberal and conservative blocks, Experiment 1.

	*d*′_conservative_		*d*′_liberal_		*c*_conservative_		*c*_liberal_	
	*M*	CI_95%_	*M*	CI_95%_	*M*	CI_95%_	*M*	CI_95%_
**Feedback**								
Patrol	0.06	0.10	0.14	0.09	0.64	0.12	-0.19	0.14
Percent	0.08	0.08	0.18	0.11	0.69	0.08	-0.59	0.10
**No feedback**								
Patrol	0.12	0.10	0.19	0.08	0.26	0.14	-0.46	0.13
Percent	0.12	0.09	0.19	0.10	0.27	0.08	-0.34	0.09

Before comparing criterion shifting between the two tasks in an ANOVA, we sought evidence of the efficacy of our shifting manipulations, i.e., that shifts were significantly greater than zero in both the Percent and Patrol tasks. We submitted values of *c* in liberal and conservative test blocks to planned paired samples *t*-tests for each of the four Task × Feedback pairings (Percent and Patrol, with and without feedback). Differences in *c* between liberal and conservative blocks were highly significant in each case (all *t*s > 7, all *p*s < 0.001).

**FIGURE 2:**
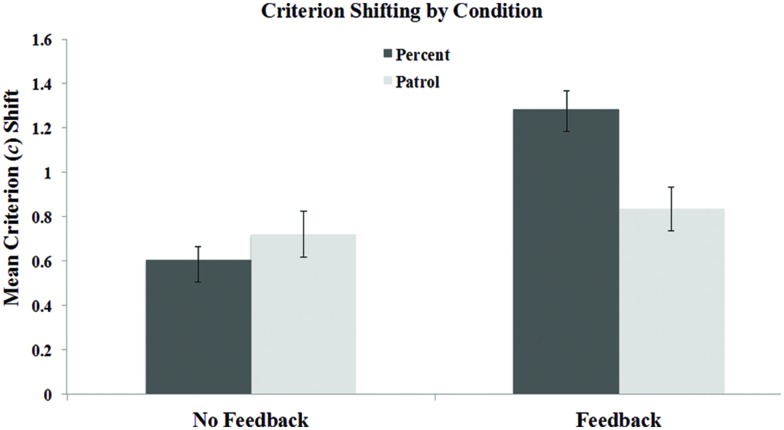
**Mean criterion shifts in the feedback and no feedback conditions of the Percent and Patrol tasks in Experiment 1.** Error bars represent the SEM.

Mean criterion shifts (*c*_conservative_ – *c*_liberal_) are displayed as a function of task and feedback condition in **Figure 2**. As is evident from the figure, criterion shifting was robust, and increased with feedback. A Task × Feedback ANOVA revealed a main effect of task, *F*(1,110) = 5.56, *p* < 0.05, $\eta_{p}^{2}$ = 0.048, and feedback, *F*(1,110) = 14.7, *p* < 0.001, $\eta_{p}^{2}$ = 0.118, indicating that participants shifted significantly more in the Percent task than in the Patrol task, and that feedback increased shifting across both tasks. The main effect of task, however, was driven by the feedback condition, reflected by a significant Task × Feedback interaction, *F*(1,110) = 15.9, *p* < 0.001, $\eta_{p}^{2}$ = 0.126. Without feedback, shifting was directionally (but non-significantly) greater in the Patrol task, *t*(54) = 1.42, *p* = 0.16, but participants receiving feedback shifted far more in the Percent task, *t*(56) = 3.91, *p* < 0.001.

**FIGURE 3:**
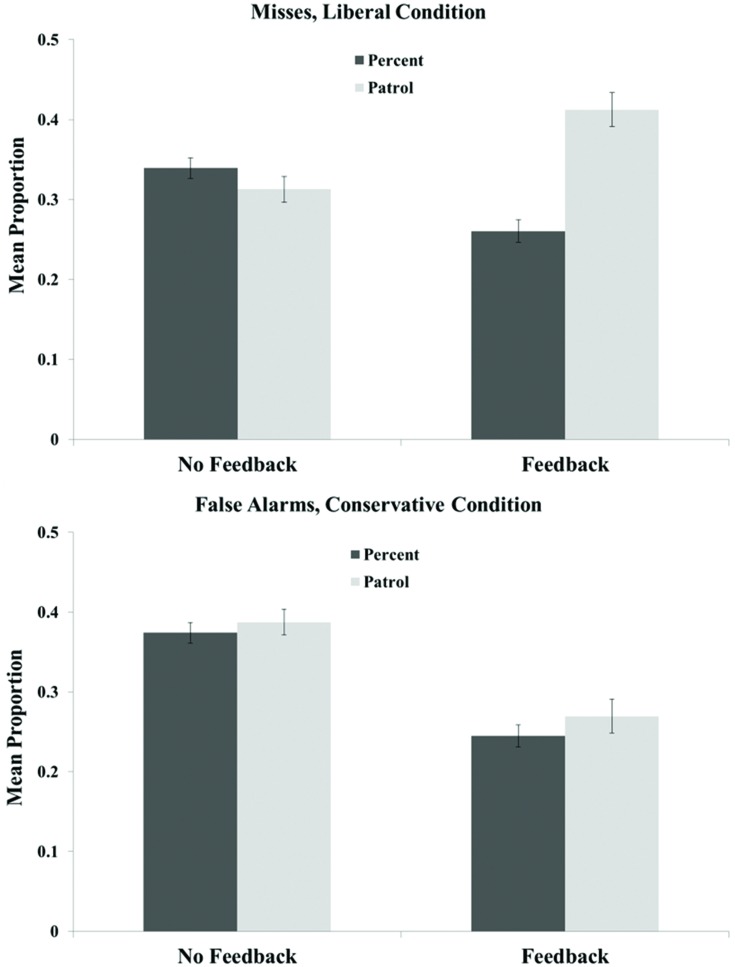
**Critical errors in liberal test blocks (misses, **top**) and conservative test blocks (false alarms, **bottom**) across recognition tasks in Experiment 1.** Error bars represent the SEM.

Criterion shifts, while substantial, were not sufficient to minimize critical errors in the Patrol task (defined as misses in the liberal condition and false alarms in the conservative condition; see **Figure 3**). Without feedback, the critical false alarm and miss rates were 0.39 and 0.31, respectively, despite the strong instructional motivation to consider such errors unacceptable. These error rates were statistically equivalent to those in the Percent condition (both *p*s > 0.18). Feedback moderately reduced critical errors in both tasks, with one exception: misses in the liberal condition were approximately 0.10 *higher* with feedback than without. Thus, liberal misses in the feedback condition were much more frequent in the Patrol than in the Percent task, *t*(56) = 6.681, *p* < 0.001. Conservative false alarms in the feedback condition were roughly equivalent across the two tasks, *t*(56) = 1.606, *p* = 0.11.

In order to confirm that greater criterion shifts are associated with reduced critical errors, we calculated the correlation of shift amount and critical error rates across all Task × Feedback conditions. Strong relationships were observed between shifting and critical false alarms, *r*(223) = -0.65, *p* < 0.001 and between shifting and critical misses, *r*(223) = -0.62, *p* < 0.001. The negative direction of these relationships indicates that participants shifting more between liberal and conservative blocks committed fewer critical errors.

The results of Experiment 1 suggest that insufficient criterion placement in recognition memory is not limited to manipulations of target probability: in the security patrol scenario, the avoidance of critical misses and false alarms was described as imperative, yet both types of errors were committed at high rates. Indeed, critical error rates in the Patrol task did not differ from those of the Percent condition, which specified no critical errors to be avoided and involved no justification for criterion shifting beyond the target base rates. As expected, trial-by-trial feedback was generally associated with larger criterion shifts and a reduction in critical errors. However, participants receiving feedback still committed such errors on approximately 25% of trials, and in the liberal condition of the Patrol task, feedback apparently made participants more conservative. Even with little or no diagnostic memory evidence to rely upon, a compelling subjective motivation to adopt a simple decision rule that would avoid critical errors, and pointed negative feedback each time such mistakes were made, participants’ rates of such errors were well above floor levels.

Why didn’t participants execute large enough criterion shifts to prevent critical errors? The ironic effect of feedback in the liberal blocks of the Patrol task may be revealing. While the base rate of old items in the Percent task shifted between 70 and 30%, the probability of a suspicious individual in the Patrol task was 30% throughout the test. As noted above, we chose this base rate in order to hold suspicious items to a minority of trials, intended as a realistic feature of a security patrol. However, feedback likely allowed participants to learn that targets were relatively uncommon (e.g., Kantner and Lindsay, 2010), countervailing the instruction to avoid misses in liberal test blocks. That participants were induced to adopt a more conservative criterion with feedback suggests that they adapted their criteria to the low probability of a target, an appropriate strategy for increasing the overall proportion of correct responses but one contrary to the central goal of minimizing misses. This possibility is consistent with work by Maddox and Bohil (1998, 2005) demonstrating that participants in a perceptual categorization task set suboptimal criteria when optimality (in maximizing financial rewards) is at cross-purposes with the maximization of accuracy.

Thus, participants appear to have prioritized attempts at accuracy over the consistent application of a task-relevant decision rule. An emphasis on accuracy in the liberal Patrol task would be consistent with hypothesis that participants fail to discount memory evidence: regardless of the extremely poor quality of the memory evidence for discriminating old and new items, participants may have persisted in using such evidence as a basis for judgments in the hope of discerning the correct response. As a result, easily avoidable critical errors were prevalent.

The Patrol task in Experiment 1 was designed to test the limits of participants’ reliance on memory evidence by producing both near-zero old–new discrimination and a clear and compelling subjective valuation on the avoidance of misses or false alarms. The finding that criterion shifts in that task were no greater than in a traditional target probability manipulation suggests a limit on criterion flexibility common to both tasks. A limitation of the Patrol task, however, is the lack of a personal consequence for critical errors. While participants clearly understood the task and shifted adaptively, they might have been induced to further constrain the use of memory evidence if they themselves were affected by their decisions. We tested this possibility by placing a financial consequence on critical errors in Experiment 2.

## Experiment 2

The simulated nature of the Patrol condition in Experiment 1 left open the possibility that participants overused memory evidence because the consequences of critical errors were fictional. We addressed this possibility in Experiment 2 by using asymmetric payoff schedules to induce criterion shifts (e.g., Healy and Kubovy, 1978). Participants studied and were tested on the same materials as in the Percent and Patrol tasks, but with no cover story or shifts in target probability. Critically, money was awarded for each correct response, while the penalty for errors varied to drive either liberal or conservative responding. Experiment 2 also included the Percent and Patrol tasks from Experiment 1. Due to concerns about the length of the experiment (especially given the extremely difficult nature of the recognition tests), each participant completed only two of the three tasks. The question of interest was whether a personal financial incentive would drive criterion shifts exceeding those of the Percent and Patrol tasks.

The Payoff condition was also valuable in assessing one potential explanation for the poor criterion placement observed in Experiment 1: perhaps participants realized that they should disregard memory evidence in making their recognition decisions and that they should instead rely on the most probable outcome/patrol directive, but chose not to because such extreme criteria entail a monotonous response pattern (i.e., nearly all “old” responses in liberal blocks and nearly all “new” responses in conservative blocks). While the desire to intermix responses alone would likely not account for the high rates of critical errors observed in Experiment 1, it is possible that some participants deliberately avoided extreme criteria for that reason, demonstrating a limit on criterion flexibility specific to very low *d*′ situations (when *d*′ is higher, by contrast, optimality does not require extreme criteria). If so, the Payoff condition should mitigate this strategy: we expect that few participants would knowingly sacrifice bonus money in exchange for a more varied response pattern.

Experiment 2 also included a slight modification to the Patrol task, designed to address the unexpected tendency for feedback to increase the miss rate in the liberal condition. As discussed above, feedback likely drove this increase by conveying the low base rate of targets, drawing participants into a conservative guessing strategy. Eliminating the diagnostic value of the base rate for guessing correctly, then, should restrict the influence of feedback to dissuading misses, reversing its effect in liberal Patrol blocks. To test this hypothesis, we set the target base rate to 0.50 in each block of the Patrol task in Experiment 2.

### Method

#### Participants

Two hundred thirty-four undergraduates at the University of California, Santa Barbara participated for course credit. The number of subjects in each of the six groups appears in **Table 2**.

**Table 2:** Mean *d*′ and criterion in conservative and liberal blocks, criterion shifting, critical false alarms, and misses in Experiment 2.

	*d′*_conservative_		*d′*_liberal_		*c*_conservative_		*c*_liberal_		Shifting		Critical FA		Critical miss	
	*M*	CI_95_	*M*	CI_95_	*M*	CI_95_	*M*	CI_95_	*M*	CI_95_	*M*	CI_95_	*M*	CI_95_
**Feedback**						
**Patrol vs. Payoff (*N* = 38)**						
Patrol	0.21	0.09	0.29	0.15	0.36	0.19	-0.63	0.18	0.99	0.36	0.35	0.06	0.25	0.04
Payoff	0.18	0.11	0.09	0.11	0.74	0.19	-0.87	0.18	1.61	0.43	0.24	0.05	0.20	0.04
**Percent vs. Payoff (*N* = 38)**						
Percent	0.09	0.13	0.14	0.12	0.84	0.17	-1.01	0.20	1.84	0.36	0.21	0.04	0.18	0.04
Payoff	0.12	0.11	0.20	0.12	0.97	0.20	-0.95	0.20	1.92	0.39	0.19	0.05	0.19	0.05
**Percent vs. Patrol (*N* = 40)**						
Percent	0.11	0.11	0.24	0.12	0.73	0.15	-0.81	0.18	1.54	0.29	0.24	0.04	0.20	0.03
Patrol	0.16	0.12	0.16	0.15	0.43	0.23	-0.82	0.19	1.25	0.40	0.35	0.06	0.22	0.04
**No Feedback**						
**Patrol vs. Payoff (*N* = 44)**						
Patrol	0.25	0.10	0.14	0.11	0.38	0.19	-0.52	0.19	0.90	0.34	0.34	0.05	0.31	0.05
Payoff	0.15	0.10	0.26	0.13	0.43	0.15	-0.53	0.16	0.95	0.28	0.32	0.05	0.29	0.05
**Percent vs. Payoff (*N* = 39)**						
Percent	0.13	0.14	0.17	0.12	0.45	0.15	-0.53	0.15	0.99	0.28	0.33	0.05	0.29	0.04
Payoff	0.20	0.11	0.20	0.10	0.43	0.17	-0.58	0.19	1.00	0.32	0.33	0.05	0.28	0.05
**Percent vs. Patrol (*N* = 35)**						
Percent	0.10	0.13	0.03	0.18	0.34	0.10	-0.37	0.11	0.71	0.17	0.36	0.04	0.36	0.04
Patrol	0.15	0.14	0.26	0.13	0.21	0.16	-0.41	0.20	0.62	0.17	0.41	0.06	0.32	0.06

#### Materials

Materials were identical to those of Experiment 1.

#### Procedure

Each participant completed two recognition study-test cycles corresponding to two of three tasks: Percent, Patrol, and Payoff. The two tasks to be completed and their order were determined randomly. The Percent task was identical to that of Experiment 1. The Patrol task was identical to that of Experiment 1 except that the base rate of old items was 0.50 (as opposed to 0.30 in Experiment 1). In the Payoff task, participants were informed that each correct response they gave would be worth 10 cents, while the penalty for errors would vary according to the location of the individuals: in liberal blocks, participants lost 20 cents for a miss and nothing for a false alarm; in conservative blocks, participants lost nothing for a miss and 20 cents for a false alarm. The base rate of old items was 0.50 in each test block. Feedback in the payoff task was identical to that of the Percent task except for the addition of the words “+10 cents” following correct responses, “-20 cents” following critical errors, and “±0 cents” following non-critical errors.

### Results and Discussion

Old–new discrimination was nearly identical to that of Experiment 1. Across all participants, the mean *d*′ was 0.146 (corresponding to a mean hit rate of 0.55 and a mean FA rate of 0.49), a value significantly greater than zero, *t*(233) = 13.772, *p* < 0.001. *d*′ scores did not vary for any of the groups as a function of task (all *p*s > 0.17) or across liberal and conservative test blocks (all *p*s > 0.32). Feedback did not affect *d*′ scores in any of the task pairings (all *p*s > 0.42).

**FIGURE 4:**
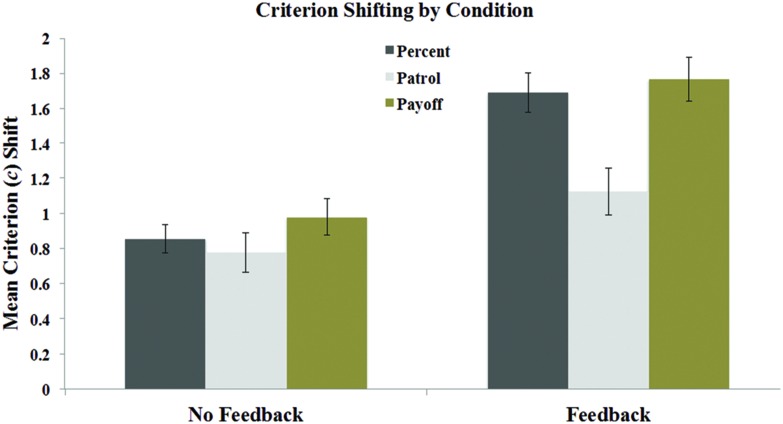
**Mean criterion shifts in the feedback and no feedback conditions of the Percent, Patrol, and Payoff tasks in Experiment 2.** Error bars represent the SEM.

**FIGURE 5:**
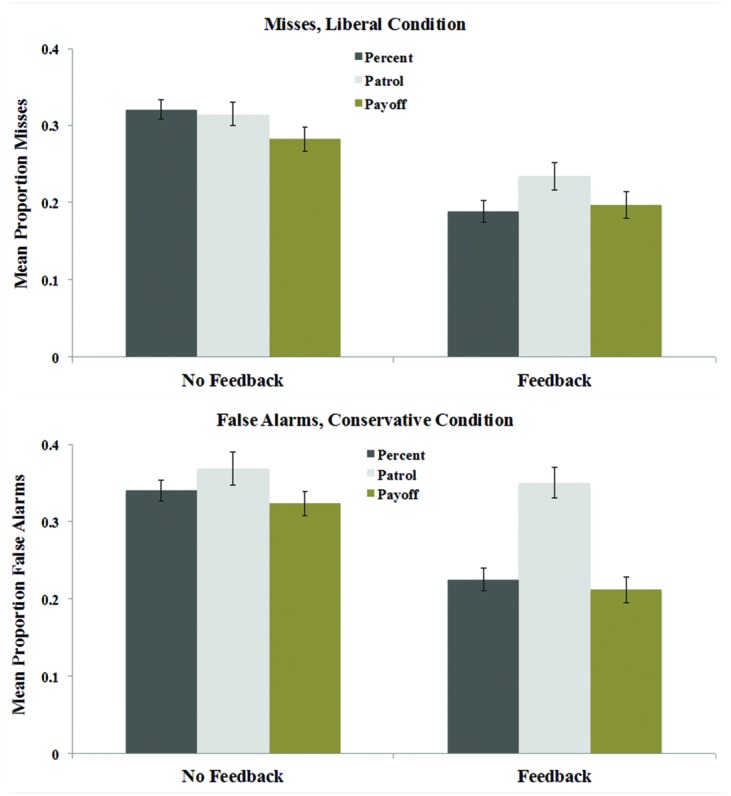
**Critical misses **(top)** and false alarms **(bottom)** across recognition tasks in Experiment 2.** Error bars represent the SEM.

The means of interest for each group are displayed in **Table 2**. Differences in *c*-values between liberal and conservative test blocks were again highly significant in each task across all groups (all *t*s > 4, all *p*s < 0.001). One-way ANOVAs revealed no significant differences in criterion values as a function of task pairing (e.g., values of *c* were the same in the Percent task across the Percent vs. Patrol and Percent vs. Payoff groups), all *p*s > 0.09. Therefore, to facilitate visual comparisons across tasks, we pooled the results for each task across groups, creating an overall mean for each task. Mean criterion shifts are displayed in **Figure 4**, and mean critical miss and false alarm rates are displayed in **Figure 5**. As is clear from these figures, criterion shifts were insufficient to eliminate critical errors, regardless of the task. Trial-by-trial feedback appeared to reduce critical errors, but not below rates of approximately 20% across tasks.

Unfortunately, because each participant completed two of the three tasks, these data do not lend themselves to an omnibus ANOVA. Therefore, we submitted the results from each of the six within-subjects task pairings (Patrol vs. Payoff, Percent vs. Payoff, and Percent vs. Patrol, each with and without feedback) to paired-samples *t*-tests with a Bonferroni-corrected alpha level of 0.0167. This alpha level is equal to 0.05/3 and reflects the fact that three *t*-tests (comparing criterion shifts, critical misses, and critical false alarms) were conducted for each of the six groups. We note that this adjusted alpha level did not affect the significance of any *t*-test result relative to the conventional 0.05 level.

In the absence of feedback, shifting was approximately equivalent in the Percent, Patrol, and Payoff conditions, yielding average critical miss and false alarm rates above 30% across tasks. There were no significant differences in shifting, critical misses, or critical false alarms in any of the cross-task comparisons. In the Percent vs. Patrol group, there was a marginal tendency for lower critical false alarms in the Percent task, *t*(34) = 1.871, *p* = 0.07. Otherwise, all cross-task comparisons in the Percent vs. Patrol, Patrol vs. Payoff, and Percent vs. Payoff groups yielded *p*-values greater than 0.16 for all dependent measures.

Participants receiving feedback tended to shift criterion *less* in the Patrol condition than in the Percent and Payoff conditions. In the Patrol vs. Payoff group, shifting was significantly greater in the Payoff task, *t*(37) = 3.337, *p* < 0.01. In the Percent vs. Patrol group, shifting was directionally but not significantly greater in the Percent task, *t*(39) = 1.490, *p* = 0.14. Shift magnitudes were roughly equivalent across the Percent and Payoff tasks, evidenced by a non-significant difference in the Percent vs. Payoff group, *p* = 0.73.

The decreased shifting in the Patrol task was accompanied by a corresponding increase in critical errors in that task relative to the Percent and Payoff tasks. In the Patrol vs. Payoff group, critical false alarms were significantly lower in the Payoff task, *t*(37) = 3.581, *p* < 0.001, while critical misses were directionally but non-significantly lower, *t*(37) = 1.549, *p* = 0.13. In the Percent vs. Patrol group, critical false alarms were significantly lower in the Percent task, *t*(39) = 4.072, *p* < 0.001, but critical misses did not differ (*p* = 0.35). Neither critical false alarms nor critical misses differed across tasks in the Percent vs. Payoff group, both *p*s > 0.27.

The results of Experiment 2 were straightforward: whether the motivation for criterion shifts was the avoidance of critical errors in a security patrol scenario, accumulation of money, or knowledge of uneven base rates, criterion placement was incommensurate with participants’ floor-level discrimination and insufficient to prevent high rates of costly errors. Moreover, shifting magnitudes were generally equivalent across the three tasks, resonant with the results of Experiment 1 in suggesting a limit to criterion flexibility that is robust across different contextual manipulations of bias. In particular, the fact that response criteria were generally no more extreme when money was at stake than when it was not suggests that inadequate criterion setting is not simply a result of participants wishing to avoid making the same response on most trials. It seems unlikely that a substantial number would have given up money to do so, especially to the extent evidenced by the critical error rates. Finally, changing the proportion of suspicious items in the Patrol task to 0.50 reversed the negative effect of feedback on critical misses observed in Experiment 1, when the prior probability was 0.30. This result supports the proposal that participants will seek out and use any information they believe can help them discern old–new status, even when doing so runs counter to the primary objective of the task.

## Experiment 3

The results of Experiments 1 and 2 suggest that limited criterion flexibility in recognition memory stems from a failure to discount memory evidence as a basis for decisions, even when memory evidence is completely ambiguous. In Experiment 3, we tested a scenario in which memory evidence was completely *absent*. Each participant completed two security patrol tasks, one of which did not include a study phase. In order to provide a reasonable context for a study-free recognition test, participants were told that while they would normally be studying pictures of suspicious individuals en route to the security patrol, their equipment malfunctioned, and they would have to complete the patrol without having studied the suspicious individuals (see Method for full instructions). In this security patrol, then, participants were fully aware that memory evidence was non-existent. If dependence on memory evidence restricts appropriate criterion shifting, then shifting in the study-free patrol should be greater than previously observed.

Our study phase-free recognition procedure is very similar to one developed by Healy and Kubovy (1978), who sought to test the hypothesis that suboptimal criterion placement arises from the memory demands of a recognition study/test procedure limiting participants’ ability to track the number of old items presented at test. In their “no memory” condition, participants received lines of dashes in place of to-be-recognized items in the study phase, followed by a standard recognition test. Participants were told that there would be correct and incorrect answers on the test, but that they would have no memory information on which to base their judgments. As in our Percent condition, participants were informed of the base rates of old items, which shifted across blocks. Healy and Kubovy (1978) compared criterion shifts in this no-memory condition with shifts in a condition containing a standard study phase and found that shifting was directionally but not significantly greater in the no-memory condition. As criterion shifts were still suboptimal in the absence of a study list, the authors interpreted these results as evidence against both the memory load hypothesis (because no items were memorized) and the hypothesis that under-shifting is driven by a failure to appreciate the distributions of old and new items (because there were no truly “old” items).

Although we used a study list-free procedure to assess a different hypothesis (i.e., that overreliance on memory evidence restricts criterion placement), Healy and Kubovy’s (1978) results suggest that participants are hard-pressed to use extreme criteria even in the absence of memory evidence. Participants may, then, embrace some other form of non-probative, internally generated decision evidence when no memory evidence is available. Although it is unclear what form this alternative evidence would take (and it may indeed be somewhat idiosyncratic), Healy and Kubovy’s (1978) results indicate that it may prevail over the consistent use of an adaptive criterion. The comparison of shifting in the study phase-free and standard Patrol tasks in Experiment 3 served as a test of this possibility. The use of more extreme criteria in the study phase-free patrol than the standard patrol would suggest the limiting influence of memory evidence overuse; if the two conditions elicit similar criteria (as in Healy and Kubovy’s data), other forms of dubious decision evidence would be implicated in poor criterion placement.

Although the lack of an encoding phase eliminates any possibility of basing decisions on memory evidence, participants might base suspicious/innocent judgments on physical characteristics of the stimuli. While no such stimulus features were in fact predictive of suspicious/innocent status, participants lacking an alternative basis for judgments might infer that certain items “look” suspicious (e.g., those wearing dark clothing), essentially treating the study-free patrol as a categorization task. To help ensure that the nature of the patrol (i.e., avoid misses or avoid false alarms) was considered the only potential basis for judgments, instructions informed participants that characteristics such as gender, skin color, clothing color, body posture, and facial expression “do not tell you anything about whether an individual is more likely to be suspicious” and that the nature of the patrol was the only basis for making judgments.

### Method

#### Participants

Ninety-one undergraduates at the University of California, Santa Barbara participated for course credit. Forty-nine completed the study-test cycle first, followed by the test with no prior study phase; the remaining 42 completed the tasks in the reverse order.

#### Materials

Materials were identical to those of Experiments 1 and 2. The assignment of suspicious vs. innocent status to test probes in the study-free patrol was functionally random for each participant.

#### Procedure

Participants completed two Patrol tests, one with a preceding study phase and one without, in a random order. Instructions were similar to those used in the Patrol task of the two previous experiments, but were adapted to provide context for the study-free patrol:

“You are currently riding in a car on the way to the security patrol. While you are on the way, you are supposed to be presented with pictures of suspicious individuals, because when you reach the security patrol, you are tested on your memory for these suspicious individuals. But there has been a problem on the way to the patrol. You were supposed to study a list of the suspicious people that you will encounter on the patrol, but your equipment malfunctioned, and you were not able to study any of these individuals before getting to the patrol.

    However, you must still complete the patrol.

    You will encounter individuals that are either suspicious or innocent, and you must decide whether each individual is suspicious or innocent, even though you did not get the chance to study the suspicious ones.”

Prior to the start of the test, participants received the following information (not included in Experiments 1 and 2):

“One piece of advice for these patrols: you will not be able to tell whether an individual is suspicious or innocent based on their gender, skin color, clothing color, body posture, or facial expression. These features do not tell you anything about whether an individual is more likely to be suspicious or more likely to be innocent.

    And because of your equipment malfunction, you did not have a chance to study the suspicious individuals earlier. The only reliable information you have in making your judgments is the knowledge of what kind of patrol you are on (city or outskirts).”

Instructions for the standard security patrol differed from the above in only two respects. First, no equipment failure was mentioned and the study phase proceeded as normal. Second, the advice given to participants prior to the test concluded with the statement “The only reliable information you have in making your judgments is whether or not you studied the individual on the way to the patrol, and the knowledge of what kind of patrol you are on (city or outskirts).”

In order to reduce the amount of instructional material to be read by participants, descriptions of the city and outskirts patrols appeared only before each type of patrol was to begin; an earlier overview included in Experiments 1 and 2 was omitted. The viewing of the city and outskirts backgrounds prior to the test was also omitted. The test procedure was identical to that of Experiments 1 and 2, except that no participants were given feedback.

### Results and Discussion

The mean *d*′ and *c*-values in each condition appear in **Table 3**. As would be expected given the lack of an encoding phase, discrimination of suspicious and innocent items was approximately zero in the study-free patrol (mean *d*′ = 0.013) and not significantly greater than chance, *p* = 0.59. *d*′ scores in the standard patrol were comparable to those of Experiments 1 and 2 (*M* = 0.179), and were again significantly greater than zero, *t*(90) = 7.215, *p* < 0.001. A 2 (Patrol Type: Standard vs. Study-Free) × 2 (Bias: Liberal vs. Conservative) × 2 (Patrol Order: Standard First vs. Study-Free First) ANOVA on *d*′-values indicated significantly better discrimination in the standard than in the study-free patrol, *F*(1,89) = 27.8, *p* < 0.001, $\eta_{p}^{2}$ = 0.238, and better discrimination among participants receiving the standard patrol first (*M* = 0.135) than among those receiving the study-free patrol first (*M* = 0.065), *F*(1,89) = 5.944, *p* < 0.05, $\eta_{p}^{2}$ = 0.063. No other significant trends were observed.

**Table 3:** Mean *d*′ and criterion values in liberal and conservative blocks, Experiment 3.

	*d*′_conservative_		*d′_liberal_*		*c*_conservative_		*c*_liberal_ x	
	*M*	CI_95%_	*M*	CI_95%_	*M*	CI_95%_	*M*	CI_95%_
**Standard patrol first**								
Standard patrol	0.19	0.07	0.25	0.09	0.33	0.11	-0.22	0.14
Study-free patrol	0.02	0.07	0.08	0.08	0.93	0.28	-0.73	0.37
**Study-free patrol first**								
Standard patrol	0.14	0.14	0.12	0.08	0.36	0.19	-0.40	0.15
Study-free patrol	0.01	0.09	-0.08	0.10	0.86	0.21	0.01	0.26

**FIGURE 6:**
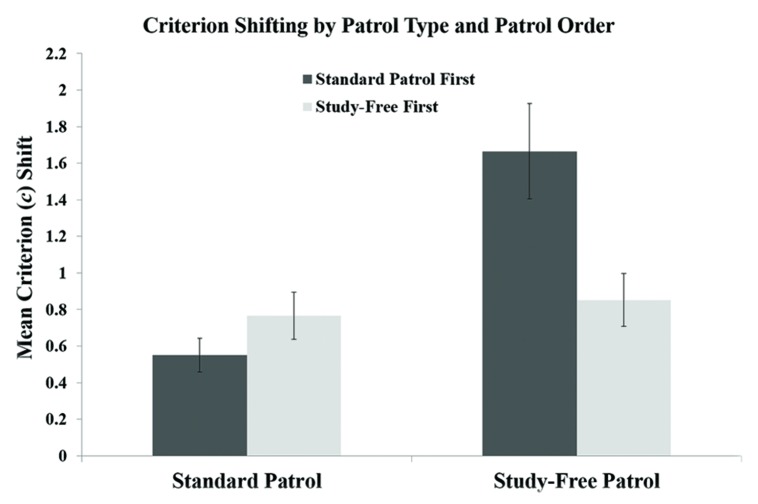
**Mean criterion shifts in the standard and study-free patrol tasks in Experiment 3.** Error bars represent the SEM.

Mean criterion shifts are presented as a function of patrol type and patrol order in **Figure 6**. As predicted, shifts were much wider in the study-free patrol than in the standard patrol, though the order of the two patrols was highly influential. When the study-free recognition task came first, shifting was similar across the two patrol types; when the standard patrol came first, shifting was approximately three times greater in the study-free patrol. A Type × Order ANOVA confirmed these trends, revealing a significant main effect of type, *F*(1,89) = 19.3, *p* < 0.001, $\eta_{p}^{2}$ = 0.178, and a significant Type × Order interaction, *F*(1,89) = 14.2, *p* < 0.001, $\eta_{p}^{2}$ = 0.137. Because patterns of criterion placement depended critically on the order of the tasks, we report critical error data separately for the study-free-first and standard-first groups.

**FIGURE 7:**
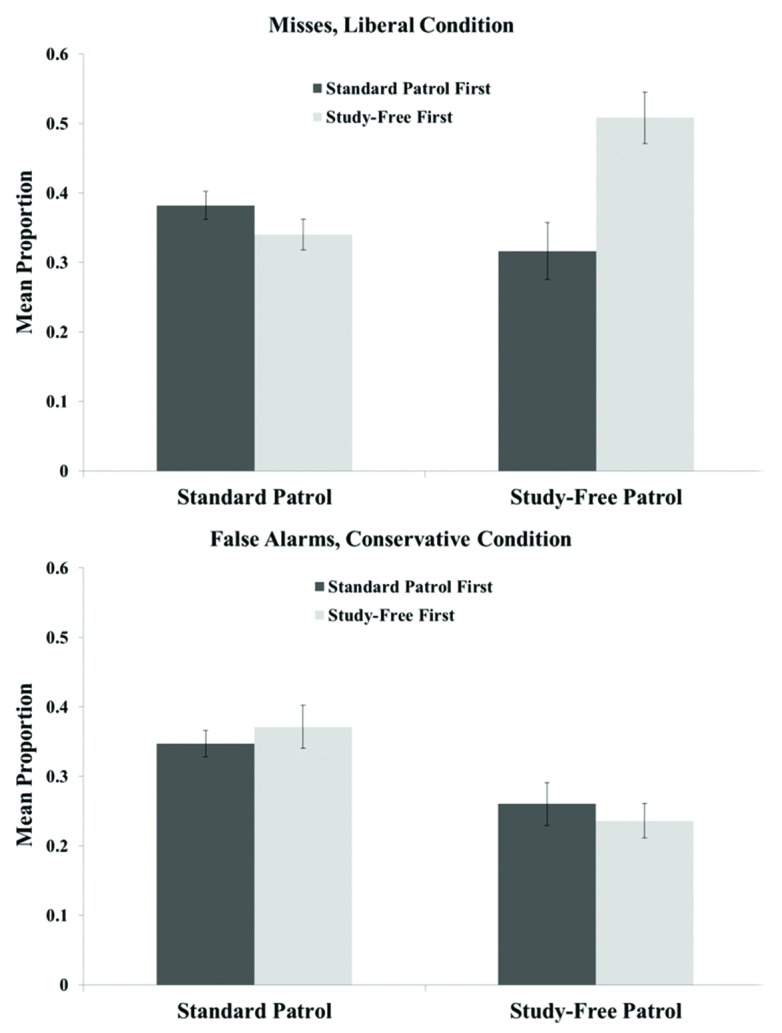
**Critical misses **(top)** and false alarms **(bottom)** in the standard and study-free patrol tasks in Experiment 3.** Error bars represent the SEM.

Critical misses and false alarms are depicted in **Figure 7**. When participants completed the study-free patrol first, critical false alarms were significantly lower in the study-free patrol than in the standard patrol, *t*(41) = 5.221, *p* < 0.001, but critical misses were significantly higher, *t*(41) = 4.514, *p* < 0.001. When the standard patrol came first, both critical misses and critical false alarms were reduced in the study-free patrol. The difference in false alarms was significant, *t*(48) = 3.158, *p* < 0.01, while the difference in misses approached but did not reach significance, *t*(48) = 1.844, *p* = 0.07.

Participants receiving the study-free patrol first were apparently reluctant to call individuals suspicious in that patrol, resulting in a high critical miss rate that rendered the mean criterion shift roughly equal to that of the standard patrol. In all other respects, the results of Experiment 3 reflect reduced critical error rates in the study-free patrol. These results support the hypothesis that criterion flexibility in recognition memory tasks is constrained by overreliance on memory evidence; when the possibility of drawing on such evidence was removed, participants increased the magnitude of shifts.

Even in the study-free patrol, however, knowledge of the proper bias was by no means the only basis for judgments: critical miss and false alarm rates were no lower than 0.32 and 0.24, respectively. These results are consonant with those of the no-memory condition of Healy and Kubovy (1978), and indicate that some other form(s) of information were frequently used to determine suspicious-innocent status. Given that memory evidence was unavailable and that participants were explicitly told to avoid basing responses on perceptual attributes of the stimuli, it is unclear how decisions against the prevailing bias were made in such cases. Informal conversations with participants at debriefing, however, suggested that many participants based judgments on perceptual stimulus attributes despite knowing that the instructions had told them otherwise. These participants sometimes reported needing some basis for their decisions, even when they had been told that would not lead to correct decisions, because they had “nothing else to go on.” Based on the results of Experiment 3, an interim conclusion is that participants in a recognition task rely on a combination of mnemonic and non-mnemonic sources of decision evidence when memory evidence is at least ostensibly available. When no form of memory evidence is available, as in the study-free patrol, participants continue to rely on non-mnemonic varieties of “evidence,” even when such evidence contains no definable signal.

**FIGURE 8:**
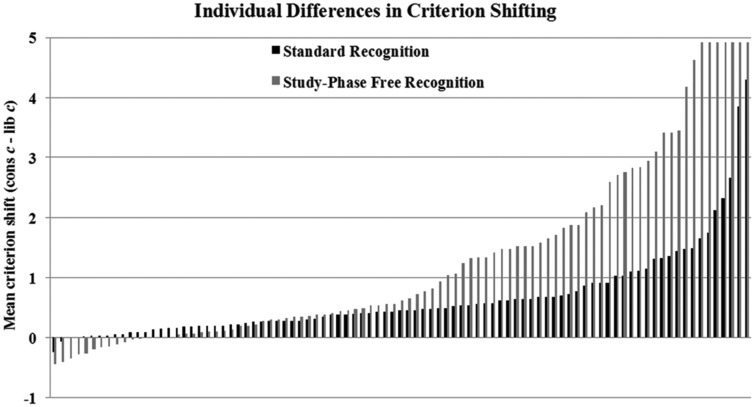
**Criterion shifts for each participant in the standard and study-free patrol tasks in Experiment 3.** In each task, the maximum shift possible was 4.91.

Removal of the study phase in the study-free patrol did not influence all participants equally, however. **Figure 8** displays criterion shifting for each participant in the standard and study-free patrols, ordered from the smallest shift for a given patrol on the left to the largest shift on the right. For example, the first pair of bars on the left represents the smallest shift by a participant in the standard patrol followed by the smallest shift by a participant in the study-free patrol; the *n*th pair of bars represents the *n*th largest shift in each task (note that shifts are ordered independently for the standard and study-free patrols, such that a given pair of bars represents the same shifting rank for each patrol type, but not necessarily the same participant). This plot illustrates a critical distinction between performance on the two patrol types: while broad individual differences characterized shifting on both tasks, many more individuals shifted at or near the maximum level (corresponding to a value of 4.91 in the present experiment) in the study-free patrol than in the standard patrol. Strikingly, 16 participants minimized critical misses, critical false alarms, or both in the study-free patrol; only two did so in the standard patrol. Removing memory evidence, then, induced many more participants to adopt an extreme criterion. Of the 16 participants eliminating critical errors in the study-free patrol, 13 had received the standard patrol first, suggesting that having experience with a standard study-test cycle first made the loss of the study phase in the study-free patrol more salient, provoking participants to abandon any attempt to utilize decision evidence. These results demonstrate that a strategy of disregarding faulty decision evidence is not beyond the means of individuals; rather, some individuals do indeed execute such shifts, while most do not.

## General Discussion

Criterion shifting in recognition memory tasks is generally inadequate, resulting in lost potential for correct responses. We tested the hypothesis, originally advanced by Green and Swets (1966), that this conservatism in criterion placement is driven in part by an overreliance on decision evidence. In order to gauge the potential for participants to disregard ambiguous decision evidence and defer to simple decision rules following from a criterion manipulation, we created a recognition paradigm in which participants had little or no recourse to memory and compelling consequences for critical errors. Criterion shifting under these conditions was insufficient to avoid striking rates of critical errors. In Experiment 1, shifting was no greater in the patrol task than in a standard target probability manipulation, and mean critical error rates were no lower than 25% even with trial-by-trial feedback admonishing participants for such errors. In Experiment 2, a payoff task with a personal financial consequence for critical errors yielded performance similar to that of the patrol and percent tasks. Even the removal of the study phase from the patrol task in Experiment 3 did not dissuade most participants from relying, in part, on information they knew was not diagnostic of target status. These findings suggest that most participants will use any information at hand to try and arrive at the correct response before they will resign themselves to a heavy reliance on a clear and adaptive decision rule, even when the evidence is transparently devoid of value.

Researchers have advanced several hypotheses regarding the basis of this limitation. These include the over/underestimation of target base rates (Ulehla, 1966), a strategy of distributing “old” and “new” responses according to the base rates (i.e., “probability matching;” Thomas and Legge, 1970), incorrect intuition as to the shape of the target and lure evidence distributions (Kubovy, 1977), and a noisy, rather than static, decision criterion (Benjamin et al., 2009). Although any of these factors may have influenced performance in the present experiments, we do not believe that they provide a compelling account of the results. First, target base rates were not manipulated in the Patrol (Experiments 1–3) and Payoff (Experiment 2) tasks, yet critical error rates were as high in these conditions as in the Percent task. These findings suggest that limits on criterion shifting are not driven by a failure to estimate or an attempt to match base rates (and estimation of the base rates was not necessary even in the Percent task). Participants could have detected and tried to match the base rates in the Patrol and Payoff tasks, but these base rates were even (except in the Experiment 1 Patrol task) and thus had no diagnostic value. Second, participants’ ability to estimate the shape of the evidence distributions would not have been a factor in the study-free Patrol task (i.e., there was no memory evidence in that task), yet most participants committed high rates of critical errors. Third, trial-by-trial variability in criterion placement has been shown to predict suboptimal shifting (Benjamin et al., 2009), but it seems unlikely that such noise would have been so great as to account for the frequency of critical errors observed in the present experiments. In addition, a criterion noise account would not explain why some participants did completely avoid critical errors, particularly in the study-free patrol (see **Figure 8**). Finally, in the “utilized” signal detection model of optimal criterion placement advanced by Lynn and Barrett (2014), suboptimality is driven by inaccurate estimates of base rates, payoffs, and/or target-lure similarity. However, our data indicate that, even jointly, these factors cannot completely account for participants’ criterion placement. Base rates and payoffs did not need to be estimated in the present experiments, and target-lure similarity was irrelevant in Experiment 3, yet most participants’ criteria did not nearly minimize critical errors or maximize payoffs.

Instead, our results support the hypothesis that most participants under-shift because they over-interpret decision evidence: apparently, people would rather attempt to be *correct* than be *correctly biased*. The present results demonstrate that even when participants are aware that the signal is effectively non-existent, context-based criterion shifts are under-utilized. We speculate that people would rather try their best to generate responses using their own sense of signal strength than simply adopt a decision rule that, in the present experiments, could have ensured their total avoidance of critical errors. With an encoding phase to draw upon (as in nearly all recognition memory experiments), participants tend to over-rely on memory evidence as the source of that signal, but even in the absence of memory evidence participants will pursue some endogenously derived “signal” as a basis for judgments. Thus, as Green and Swets (1966) described, participants refuse to “turn their backs” on the visual signal and defer to context-appropriate bias. Such an account would explain why shifting in the Percent task was at least equal to that in the Payoff and Patrol tasks despite lacking the motivators inherent to those tasks. In the Percent condition, the base rates provided a basis for guessing the correct answer; in this condition only, being correct and being correctly biased were one and the same. In the Patrol condition, by contrast, even a rebuke following a critical false alarm did not provide diagnostic information that the participant could use to discern probe status on subsequent trials.

Because most participants in Experiment 3 failed to use extreme response criteria whether or not they participated in a study session prior to test, we proposed that they based a substantial portion of their judgments on forms of evidence orthogonal to the task, such as perceptual stimulus characteristics like skin color, clothing, and gender (even though they were informed that these features were balanced across suspicious and innocent individuals). This notion may seem at odds with a signal detection theory characterization of recognition memory, which includes a unidimensional (mnemonic) evidence axis. Indeed, most models of recognition memory incorporate this unidimensional evidence assumption and are not equipped to describe performance in the near-zero *d*′ scenarios tested here. However, the dynamics of decision making under these circumstances might be described by such models with an extension to a multidimensional signal detection framework (Ashby and Soto, 2015). Multidimensional signal detection theory can be applied to situations in which stimuli may be judged according to more than one dimension, and it is often used with perceptual classification tasks in which two stimulus features jointly determine category membership.

**FIGURE 9:**
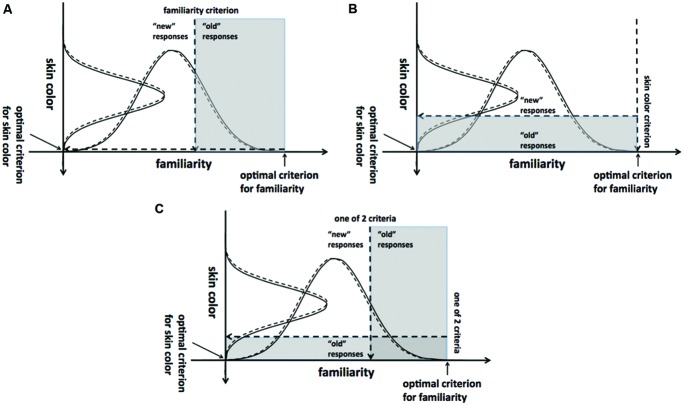
**Illustration of decision making in Experiment 3, study-free security patrol, from a multidimensional signal detection theory perspective**. Dashed lines represent noise distributions; solid lines represent signal distributions. **(A)** Test probes are called “suspicious” if their familiarity exceeds a criterial level. **(B)** Test probes are called “suspicious” if the darkness of their skin exceeds a criterial level. **(C)** Either familiarity or skin color may be used to make judgments.

We illustrate how this framework might be applied to recognition decisions under ambiguous (or absent) memory evidence in **Figure 9**, using the conservative, study-free security patrol paradigm from Experiment 3 as an example. In this patrol, subjects are instructed to avoid false alarms at all costs; between these instructions and the absence of memory evidence, the most appropriate response is to say “innocent” on every trial, yet most subjects’ false alarm rates were still quite high. **Figure 9** depicts three possible explanations for this behavior within a multidimensional signal detection framework. For simplicity, each scenario assumes that recognition decisions can be made according to a test probe’s position on a memory (familiarity) evidence axis or a perceptual (skin color) evidence axis; however, in practice any number of dimensions may become relevant for classifying a probe as recognized. Panel A depicts the use of familiarity as a basis for judgments in the study-free patrol. Without a study session, there is clearly no memory or familiarity signal relevant to the recognition decision. However, even novel items will carry some familiarity based on extra-experimental associations and encounters with previous test items. Given that subjects were instructed to make a memory judgment even with no prior study session, they may still have felt compelled to make a judgment according to the familiarity dimension. Alternatively, subjects may have ignored the instructions not to make a judgment on the basis of a perceptual characteristic (Panel B). The use of skin color as a basis for determining whether or not a test item was a suspicious individual could also lead to a high proportion of false alarms. Finally, subjects may have been using more than one dimension to decide (Panel C). For example, subjects may have responded “suspicious” if the test figure seemed familiar despite the lack of a study session or if the skin color was dark enough to surpass a criterion along that dimension. As noted above, any number of dimensions not depicted may also have been used by subjects. The use of any such dimensions as an alternative to an appropriate decision criterion will lead to an increase in critical errors.

While the group average criterion shifts in the present experiments indicate a general limit on criterion flexibility, shifting was marked by broad inter-individual variability. While we displayed this variability only for Experiment 3 (see **Figure 8**), it was robust in each experiment: across the 468 tests completed by participants in Experiment 2, for example, shifting ranged from maximal (Δ*c* > 3.5, *N* = 22) to minimal (Δ*c* < 0.10, *N* = 46). Such variability raises intriguing questions as to the factors that led some participants to shift widely and others minimally. In Experiment 3, for example, 18 participants achieved either critical miss or critical false alarm rates close to 0 – what led this subset of participants to set aside decision evidence as a basis for judgments while most relied on it? The results of Experiment 3 indicate that two aspects of the task drove many of these participants to use maximal criteria. First, 16 of the 18 cases occurred in the study-free recognition test, suggesting that removing the possibility of memory evidence is enough to convince some participants to there is no basis for judgment beyond context. Second, 13 of the 16 study-free cases occurred when a complete study-test patrol cycle preceded the study-free patrol. This finding indicates that individuals are more likely to respond appropriately in a zero-evidence scenario if they have had experience with a version of the task in which decision evidence (however faulty) was available.

The question remains, however, as to why these elements of the task induced some participants and not others to adopt extreme criteria. One possibility is that stable individual differences underlie shifting tendencies. Aminoff et al. (2012) demonstrated wide variability in criterion shifting and considered willingness to shift criterion as a latent variable (see also Kantner and Lindsay, 2012, 2014). Aminoff et al. (2012) examined a number of personality characteristics that seemed to mediate such willingness, including the BAS Fun Seeking subscale (Carver and White, 1994). Future efforts to identify the cognitive and personality correlates of criterion flexibility could help reveal the basis of individual differences in adaptive shifting behavior.

Our initial hypothesis was that the security patrol scenario would in effect restrict the range of individual differences observed in shifting by compelling participants to adopt extreme response criteria. This hypothesis was disconfirmed, suggesting a deep resistance to using extreme criteria. However, these results do not rule out the possibility of a more effective security patrol that could overcome this resistance. For example, participants in the present experiments were not informed of the reason studied individuals were considered suspicious. While the instructions in the liberal and conservative conditions implied serious consequences for critical errors, reference to a specific crime (e.g., murder) might invest participants more fully in avoiding those errors. Perhaps more significantly, the security patrol was framed instructionally as a test of memory (this was less the case in the study-free patrol, but instructions in that task mentioned that memory would have been part of the decision process had the study phase been possible). People might simply be unwilling or unable to limit their use of memory under task demands that emphasize recognition. Indeed, such a predisposition would be adaptive as long as memory evidence carried some diagnostic value, and may be difficult to “turn off” in the relatively rare cases where it does not. A security patrol paradigm that emphasizes decision making at test (rather than recognition *per se*) and that characterizes memory as one source of information toward that end (rather than an end goal in itself) might engender more effective criterion placement.

## Conclusion

We note that while participants often appear to utilize highly dubious forms of decision evidence in service of recognition judgments, adaptive criterion shifting was by no means absent in the present experiments. Shifting was, in fact, substantial in every condition of the present experiments, reducing critical errors in the Patrol and Payoff tasks below chance levels even though old–new discrimination was at chance. In Experiment 2, the Percent task with feedback yielded an overall percent correct of 64%; based on discrimination alone, it would have been close to 50%. Thus, these results point to the value of criterion shifting in improving decision making, and of further investigation into the locus of shifting tendencies, especially as a cognitive aid for memory-impaired populations (e.g., Beth et al., 2009). At the same time, they demonstrate a psychological basis for a ceiling on human flexibility in decision strategies: the desire to arrive at one’s own conclusion, however inconclusive the evidence.

## Author Contributions

All authors developed the study concept and design and contributed to the interpretation of the results. JK performed data collection and data analysis, and drafted the paper. JV and MM provided critical manuscript revisions. All authors approved the final version of the paper for submission.

## Conflict of Interest Statement

The authors declare that the research was conducted in the absence of any commercial or financial relationships that could be construed as a potential conflict of interest.
